# Is early or late biological maturation trigger obesity? A machine learning modeling research in Turkey boys and girls

**DOI:** 10.3389/fnut.2023.1139179

**Published:** 2023-02-14

**Authors:** Mehmet Gülü, Fatma Hilal Yagin, Hakan Yapici, Khadijeh Irandoust, Ali Ahmet Dogan, Morteza Taheri, Ewa Szura, Magdalena Barasinska, Tomasz Gabrys

**Affiliations:** ^1^Department of Coaching Education, Faculty of Sport Sciences, Kirikkale University, Kirikkale, Türkiye; ^2^Department of Biostatistics, and Medical Informatics, Faculty of Medicine, Inonu University, Malatya, Türkiye; ^3^Department of Sport Sciences, Imam Khomeini International University, Qazvin, Iran; ^4^Faculty of Physical Education and Sport Sciences, University of Tehran, Tehran, Iran; ^5^Department Health Sciences and Sport Sciences, University of Applied Sciences, Nysa, Poland; ^6^Department of Health Sciences, Jan Dlugosz University, Czestochowa, Poland; ^7^Department of Physical Education and Sport, Faculty of Education, University of West Bohemia, Pilsen, Czechia

**Keywords:** adolescent, childhood, puberty, overweight, body mass index, noncommunicable diseases

## Abstract

Biological maturation status can affect individual differences, sex, height, body fat, and body weight in adolescents and thus may be associated with obesity. The primary aim of this study was to examine the relationship between biological maturation and obesity. Overall, 1,328 adolescents (792 boys and 536 girls) aged 12.00 ± 0.94–12.21 ± 0.99 years, respectively (measured for body mass, body stature, sitting stature). Body weights were deter-mined with Tanita body analysis system and adolescent obesity status was calculated according to the WHO classification. Biological maturation was determined according to the somatic maturation method. Our results showed that boys mature 3.077-fold later than girls. Obesity was an increasing effect on early maturation. It was determined that being obese, overweight and healthy-weight increased the risk of early maturation 9.80, 6.99 and 1.81-fold, respectively. The equation of the model predicting maturation is: Logit (*P*) = 1/(1 + exp. (− (−31.386 + sex-boy * (1.124) + [chronological age = 10] * (−7.031) + [chronological age = 11] * (−4.338) + [chronological age = 12] * (−1.677) + age * (−2.075) + weight * 0.093 + height * (−0.141) + obesity * (−2.282) + overweight * (−1.944) + healthy weight * (−0.592)))). Logistic regression model predicted maturity with 80.7% [95% CI: 77.2–84.1%] accuracy. In addition, the model had a high sensitivity value (81.7% [76.2–86.6%]), which indicates that the model can successfully distinguish adolescents with early maturation. In conclusion, sex and obesity are independent predictors of maturity, and the risk of early maturation is increased, especially in the case of obesity and in girls.

## 1. Introduction

Childhood obesity is one of the most serious public health challenges of the 21st century (1). Overweight and obesity are defined as “abnormal or excessive fat accumulation that presents a risk to health” (1). Overweight and obesity are associated with metabolic diseases that increase the risk of noncommunicable diseases such as cardiovascular disease and diabetes (2, 3). Childhood or adolescence obesity is associated with higher risk of weight-related morbidity and premature death in adulthood (4, 5). Overweight and obesity cause at least 2.6 million deaths each year (1). The World Obesity Federation reports that there has been a dramatic increase in childhood overweight and obesity over the past 30 years (6). The overall prevalence rates of overweight/obesity and obesity in Azores adolescents were 31 and 27%, respectively (7). In a study conducted with adolescents in Turkey, the prevalence rate of overweight was found to be 18.2% (8).

Hormonal changes during puberty affect weight gain and body weight. Therefore, puberty leads to changes in body weight. These changes include changes in the amount and distribution of adipose tissue, lean body weight, and bone structure. In this period, besides rapid height increase in both sexes, weight gain also occurs (9). Childhood overweight and obesity can lead to significant health problems in adulthood; these diseases are diabetes; musculoskeletal disorders, especially osteoarthritis; cardiovascular diseases (mainly heart disease and stroke); and some types of cancer (endometrial, breast, and colon) (1). Excess adipose tissue causes oxidative stress, inflammation, apoptosis and mitochondrial dysfunctions (10, 11). That’s why, obesity may cause to the onset of type-2-diabetes, liver steatosis, neurodegenerative and cardiovascular diseases that may thrive early in lifespan (12–17).

Biological maturation is a lifespan natural process which promotes morphophysiological changes in human (18). The onset of puberty in girls is relationship with an improve in the quantity of fat mass, as a consequence of improved blood concentration of estradiol (19). According to a study, the relative age effect was found to primarily affect the U13 and U15 categories in body composition (20). In a study, a relationship was found between biological maturation and muscle strength (21). Early sexual maturation is relationship with excessiveness body weight in girls and more stature for age in both sexes (22). There are clearly dissimilarity between boys and girls in fat mass and distribution, particularly in adolescence period (23). In girls, there is proof which early sexual maturation is relationship with a more prevalence of overweight and obesity (24, 25). The number of studies in boys is quite scarce, and the evidences are mixed (25, 26).

The prevalence of obesity in children and adolescents in the United States is ~17%, posing a risk to health status and life expectancy in adulthood (27, 28). Obese children and adolescents are 5 times more likely to become obese in adulthood than non-obese children and adolescents (29). About 55% of obese children become obese in adolescence, about 80% of obese adolescents will be obese in adulthood, and about 70% will be obese over the age of 30 (29). Overweight and obesity and related diseases may be largely prevented (1). Therefore, research should focus on reducing and preventing obesity in children and adolescents. Prevention of childhood and adolescent obesity therefore needs high priority. Unlike adults, children do not have the opportunity to choose the environment they live in, the foods they consume, and the choices they make. For this reason, the growth and development of children can be affected by many factors. These factors can also cause the child or adolescent to become obese. Biological maturation status may effect individual differences, gender, height, body fat and body weight (7). Early sexual maturation is relationship with overweight (girls only) and stature in children aged 8–14 years (22). The primary aim of this study was to examine the relationship between biological maturation and obesity.

## 2. Methods

### 2.1. Participants

The design of this study was cross-sectional. The research was conducted in Kirikkale province of Turkey. There are 8,758 students between the ages of 10–13 in Kirikkale province. G*power software was used to determine the sample size of the study (30). As a result of the power analysis (alpha value = 0.05 and 1-beta value = 0.80, *η*_p_^2^ = 0.25), it was found that at least 179 should be included in the study. In this study, 1,328 participants (792 boys; age = 12.00 ± 0.94 years and 536 girls; age = 12.21 ± 0.99 years) were randomly selected. The inclusion criteria of the participants were to attend physical education classes regularly for 2 h 1 day a week. Regarding dietary attitudes, the researchers did not gather any data. Each teenager and their parents received information about the study’s methods and potential hazards before becoming participants. They were informed about the study and given the option to voluntarily select whether to take part.

### 2.2. Procedures

Permissions were obtained from public and private institutions for the study. Children, their parents and physical education teachers were informed about the measurement protocols and the purpose of the study. The information form about the research was read and signed by the parents. A form file was created for each of the participants who wanted to take part in the research voluntarily. The study was ap-proved by Kirikkale University Non-Interventional Research Ethics Committee (date: 2022-06-08, no: 2022/10) and was conducted according to the principles stated in the Declaration of Helsinki. Anthropometric measurements were taken by experts in the field. None of the children participating in the measurements were excluded from the study. Within the scope of the study, each child’s age, gender, height, body weight, leg length and sitting height measurements were taken. It was determined based on the references in the WHO child and adolescent weight classification table to determine information on obesity status.

### 2.3. Measurements

#### 2.3.1. Anthropometric measurements

Standardized procedures were applied for the anthropometric measurements of the participants (31). Height and sitting heights were measured with a 0.1 cm long portable stadiometer (Seca 213, Hamburg, Germany). Tanita Body Composition Analyzer device (Tanita, BC-418, Japan) was used for body weight assessment. BMI value calculation was obtained by dividing body weight (kg) by the square of body height (m). To determine the maturity status of the participants, the percentage of estimated adult height (%PAS) included at the time of observation was estimated using (32). In determining the maturity level of each participant, classification was made according to the %PAS z-score. Subsequently, the maturation status of the participants was classified as early (z-score > 0.5), timely (z-score ± 0.5), and late (z-score < 0.5).

#### 2.3.2. Sitting height measurement

The sitting heights of the participants were measured with a Holtain brand (stadiometer with 0.1 mm precision) measuring device. After the chair was adjusted according to the height of the participant, they were asked to take a deep breath and sit upright without moving. The resulting value was recorded in centimeters.

#### 2.3.3. Somatic maturation

Estimated adult body height (PAS) was used as an indicator of maturity (Khamis and Roche, 1994). Height was determined and treated as the estimated percentage of adult height (%PAS). PAS protocol calculation was calculated by age (decimal), body mass, height and mean parental height. The heights of the parents were collected with an informed consent form. The PAS variable was expressed as the percentile of estimated adult height (APAS) (33). Among children of the same chrono-logical age, individuals with a higher estimated adult height were considered to be in a more advanced state of physical maturation compared with shorter individuals (Khamis and Roche, 1994). The Khamis-Roche method has been applied in many studies in order to predict the biological maturity status (34, 35). In this study, a grouping was made among children. Using the sample median z-score of the obtained %PAS value, the latest maturation status (*p* < 50%) and the earliest maturation status (*p* > 50%) are given.

#### 2.3.4. Obesity classification

The prevalence of overweight and obesity in adolescents is defined according to the WHO growth reference for school-aged children and adolescents (overweight = 1 standard deviation body mass index for age and sex, and obese = 2 standard deviations body mass index for age and sex) (1). In this direction, body mass index (BMI) values were calculated by measuring the height and weight of the individuals; The 85th and 95th percentiles were considered overweight, and those above the 95th percentile were considered obese (36) determining the BMI values and obesity status of the participants, the child body mass index calculation application on the website of Centers for Disease Control and Prevention (CDC) was used (37).

#### 2.3.5. Data analysis

The suitability of the quantitative variables to the univariate normal distribution was examined by visual (histogram and probability graphs) and analytical (Shapiro–Wilk test) methods. The Henze-Zirkler test was used to examine the multivariate normal distribution. The assumption of homogeneity of variances was examined with the Levene test. Since quantitative variables were not normally distributed, they were ex-pressed as median, and interquartile range (IQR). The two-way PERMANOVA (Permutational Analysis of Variance) test, with the Euclidean distance as the similarity matrix as the first factor maturity groups and the second factor obesity groups, was used to examine the difference and interaction effect between the groups (Permutation *N* = 9,999). In multivariate analysis, possible risk factors were examined by binary logistic regression using independent predictors in early and late maturity. Hosmer-Lemeshow and Omnibus tests were used to evaluate the logistic regression model and its coefficients. Classification performance measures were calculated using the confusion matrix regarding the prediction performance of the logistic regression model. In evaluating the performance of the model, accuracy, F1-score, sensitivity, specificity, positive predictive value, negative predictive value criteria were used. A *p* < 0.05 was considered statistically significant in all results. American Psychological Association (APA) 6.0 style was used to report statistical differences (38). Statistical analyzes were performed using Python 3.9 software and SPSS 28.0 (IBM Corp., Armonk, NY, United States) package program. GraphPad 9.4.1 program was used for graphics.

## 3. Results

**Figure 1:**
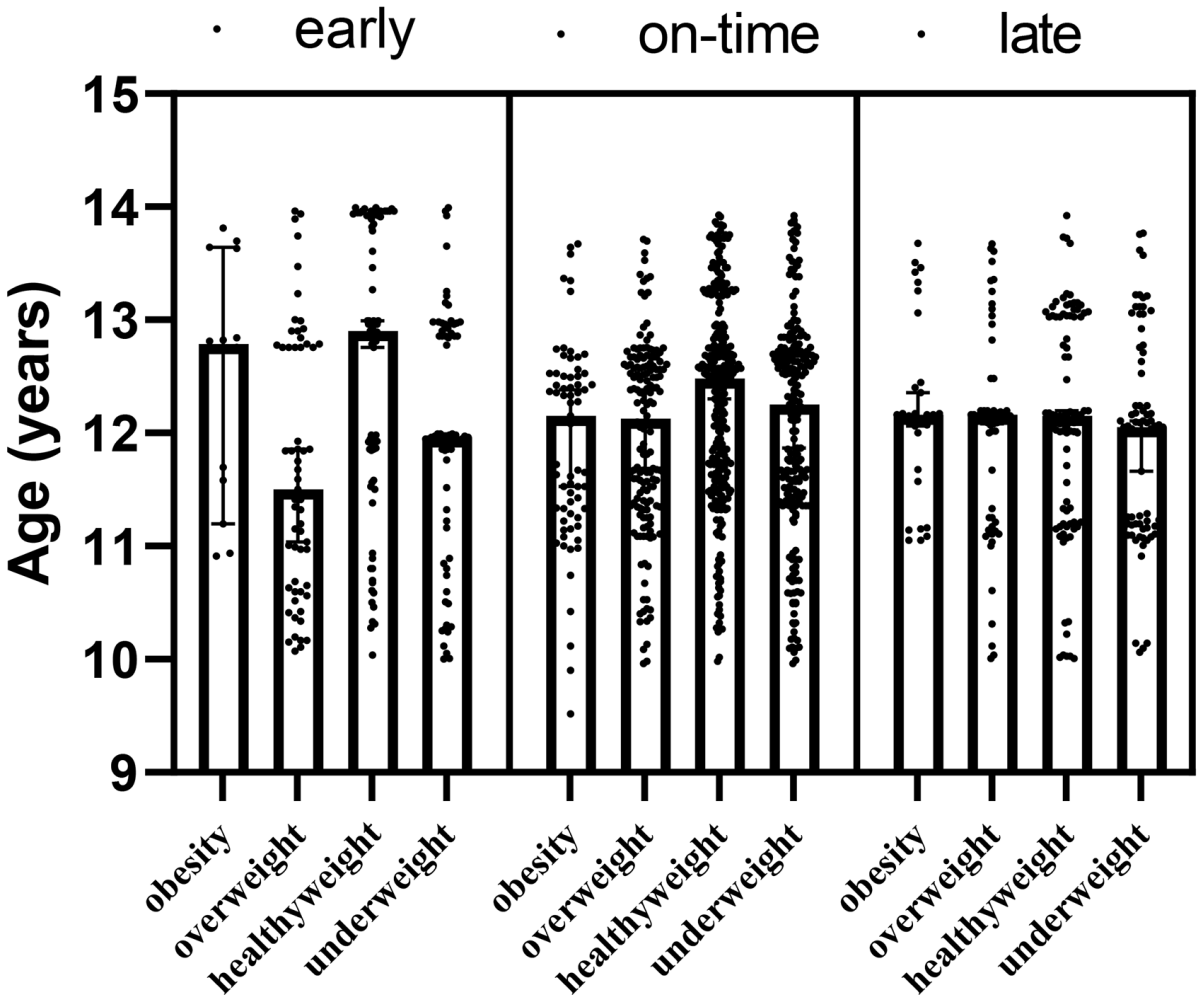
Ages of the participants by maturity and obesity groups.

The results of PERMANOVA showed that besides the interaction effect of maturity × obesity (*F* = −81.539; *p* < 0.001) at the age of the participants, obesity was the main effect (*F* = 8.089; *p*_2_ < 0.001). Interaction results showed that those with early maturation and healthyweight were significantly older. However, the main effect of maturity was not significant (*F* = 0.841; *p*_1_ = 0.240) (Table 1; Figure 1).

**Table 1:** Changes in the age of the participants according to the maturity and obesity groups.

Groups	Median (IQR)	Maturity main effect	Obesity main effect	Interaction
		*F* value	*F* value	
		*p*_1_ value	*p*_2_ value	
Early-obesity	12.78 (2.05)	*F* = 0.841 *p*_1_ = 0.240	*F* = 8.089 *p*_2_ < 0.001	*F* = −81.539 *p* < 0.001
Early-overweight	11.50 (2.15)			
Early-healthyweight	12.90 (2.07)			
Early-underweight	11.96 (1.62)			
On-time-obesity	12.15 (1.23)			
On-time-overweight	12.10(1.25)			
On-time-healthyweight	12.48 (1.22)			
On-time-underweight	12.25 (1.26)			
Late-obesity	12.15 (1.23)			
Late-overweight	12.10 (1.25)			
Late-healthyweight	12.48 (1.22)			
Late-underweight	12.25 (1.26)			
*Post-hoc* analysis for obesity factor		Obesity-healthyweight *p*_3_ = 0.016		
		Overweight-healthyweight *p*_3_ < 0.001		
		Healthy weight-underweight *p*_3_ < 0.001		

IQR, interquartile range; *p*_1_ value, significance test result between maturity groups; *p*_2_ value, significance test result between obesity groups; *p*_3_ value, the results of the in-group comparison significance test.

**Figure 2:**
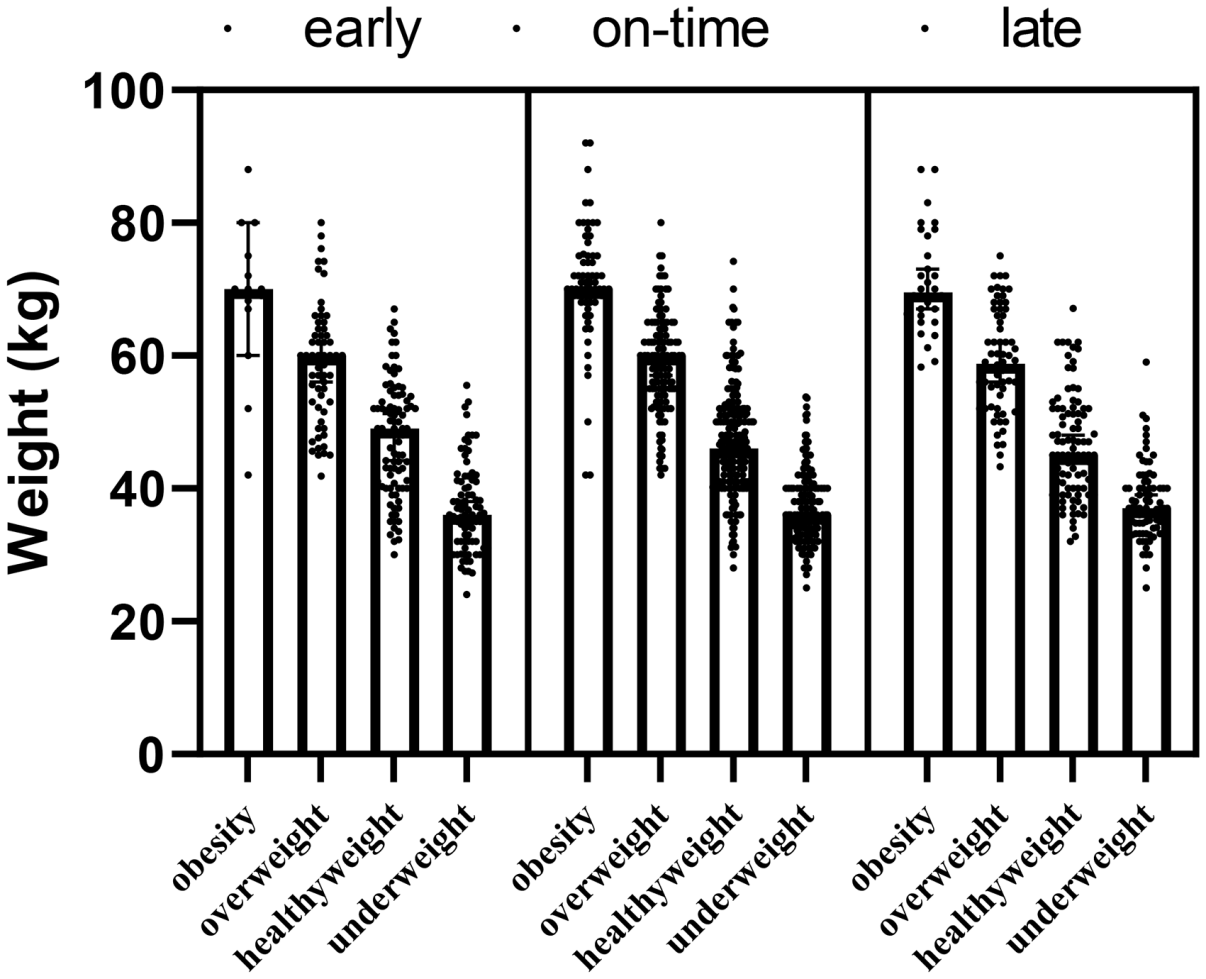
The body weights of participants according to maturity and obesity groups.

In addition to the main effect of maturity (*F* = 3.682; *p*_1_ = 0.002) and obesity (*F* = 529.580; *p*_2_ < 0.001) groups for weight, the interaction effect of maturity x obesity (*F* = −84.307; *p* = 0.02) was significant (Table 2; Figure 2).

**Table 2:** Changes in the body weight of the participants according to the maturity and obesity groups.

Groups	Median (IQR)	Maturity main effect	Obesity main effect	Interaction
		*F* value	*F* value	
		*p*_1_ value	*p*_2_ value	
Early-obesity	70 (8)	*F* = 3.682 *p*_1_ = 0.002	*F* = 529.580 *p*_2_ < 0.001	*F* = −84.307 *p* = 0.02
Early-overweight	60 (11.8)			
Early-healthyweight	49 (13.6)			
Early-underweight	36 (9.1)			
On-time-obesity	70 (7)			
On-time-overweight	60 (9)			
On-time healthyweight	46 (9.95)			
On-time-underweight	36 (6.25)			
Late-obesity	70 (7)			
Late-overweight	60 (9)			
Late-healthyweight	46 (9.95)			
Late-underweight	36 (6.25)			
*Post-hoc* analysis for obesity factor		Obesity-overweight *p*_3_ < 0.001		
		Overweight-healthyweight *p*_3_ < 0.001		
		Healthy weight-underweight *p*_3_ < 0.001		
		Overweight-healthyweight *p*_3_ < 0.001		
		Healthy weight-underweight *p*_3_ < 0.001		
*Post-hoc* analysis for maturity factor		Early-on time *p*_3_ = 0.48		
		Early-late *p*_3_ = 0.046		
		On time-late *p*_3_ = 0.09		

IQR, interquartile range; *p*_1_ value, significance test result between maturity groups; *p*_2_ value, significance test result between obesity groups; *p*_3_ value, the results of the in-group comparison significance test.

**Figure 3:**
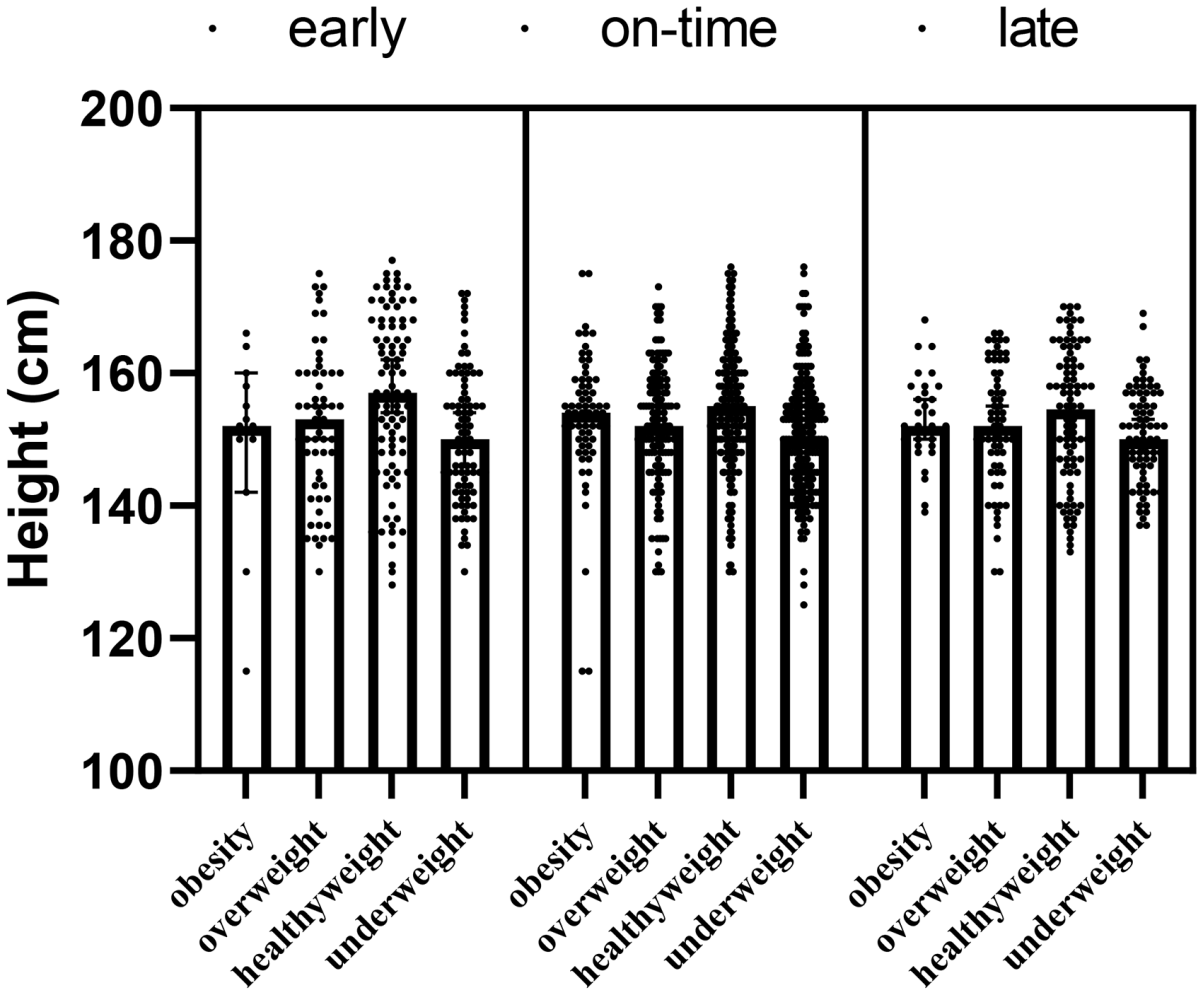
The height of participants according to maturity and obesity groups.

The effect of maturity x obesity (*F* = −84.934; *p* = 0.043) was significant for the height of the participants, and although obesity was the main effect (*F* = 9.341; *p*_2_ < 0.001), there was no main effect for maturity (*F* = 0.380; *p*_1_ = 0.521), and our interaction results showed that those with healthyweight and early maturity had a higher height (Table 3; Figure 3).

**Table 3:** Changes in the height of the participants according to the maturity and obesity groups.

Groups	Median (IQR)	Maturity main effect	Obesity main effect	Interaction
		*F* value	*F* value	
		*p*_1_ value	*p*_2_ value	
Early-obesity	152 (8)	*F* = 0.380 *p*_1_ = 0.521	*F* = 9.341 *p*_2_ < 0.001	*F* = −84.934 *p* = 0.043
Early-overweight	153 (17)			
Early-healthyweight	157 (19)			
Early-underweight	150 (14.5)			
On-time-obesity	154 (9)			
On-time-overweight	152 (10)			
On-time-Healthyweight	155 (10.5)			
On-time-underweight	150 (11.5)			
Late-obesity	154 (9)			
Late-overweight	152 (10)			
Late-healthyweight	155 (10.5)			
Late-underweight	150 (11.5)			
*Post-hoc* analysis for obesity factor			Obesity-underweight *p*_3_ = 0.013	
			Overweight-underweight *p*_3_ = 0.015	
			Healthyweight-underweight *p*_3_ < 0.001	

IQR, interquartile range; *p*_1_ value, significance test result between maturity groups; *p*_2_ value, significance test result between obesity groups; *p*_3_ value, the results of the in-group comparison significance test.

**Figure 4:**
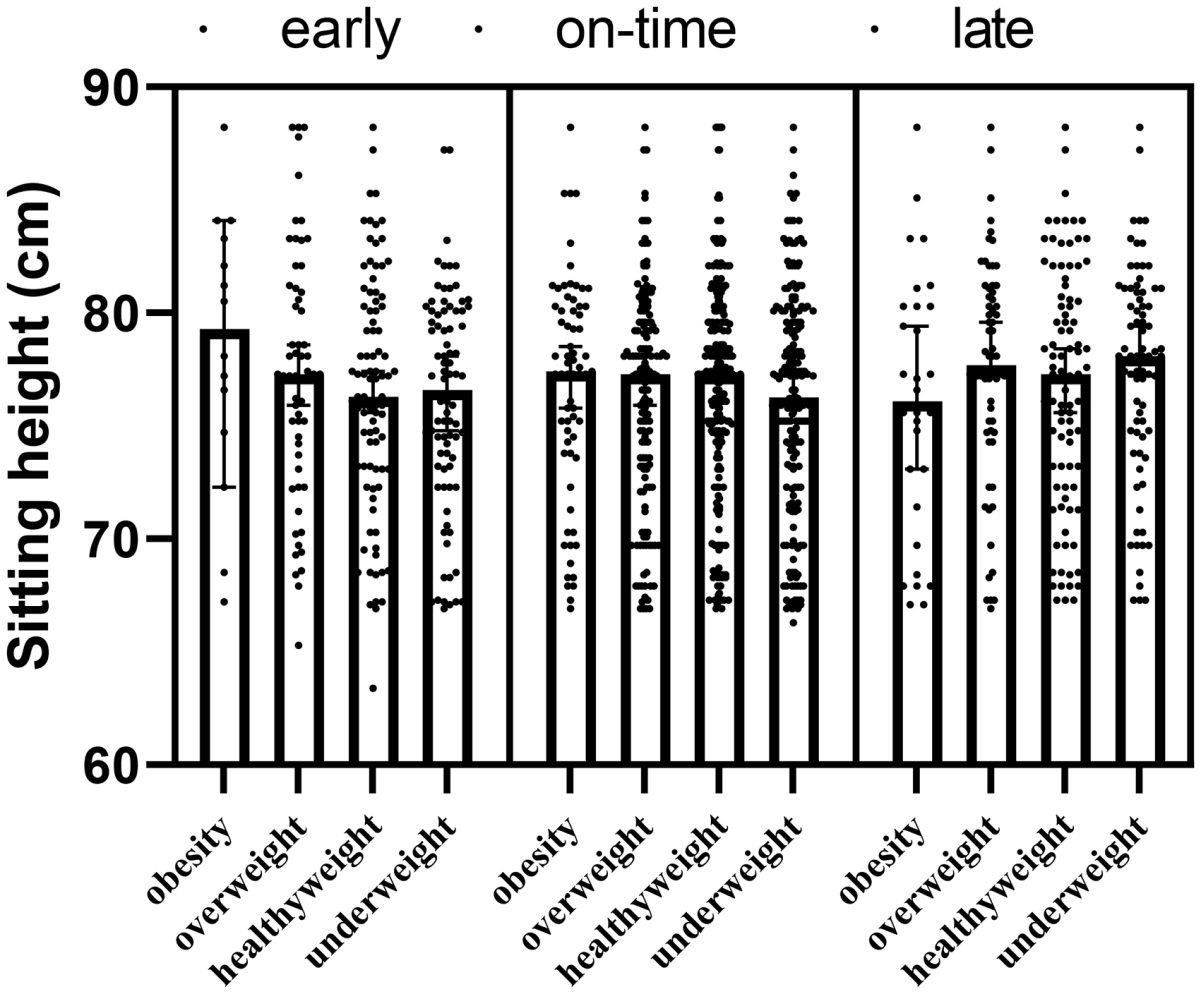
The sitting height of participants according to maturity and obesity groups.

There was no interaction effect of maturity × obesity (*F* = −86.300; *p* = 0.054) on sitting height of the participants, also maturity (*F* = 0.981; *p*_1_ = 0.195) and obesity (*F* = 0.232; *p*_2_ = 0.763) had no main effects (Table 4; Figure 4).

**Table 4:** Changes in the sitting height of the participants according to the maturity and obesity groups.

Groups	Median (IQR)	Maturity main effect	Obesity main effect	Interaction
		*F* value	*F* value	
		*p*_1_ value	*p*_2_ value	
Early-obesity	79.3 (8.6)	*F* = 0.981 *p*_1_ = 0.195	*F* = 0.232 *p*_2_ = 0.763	*F* = −86.300 *p* = 0.054
Early-overweight	77.3 (7.4)			
Early-healthyweight	76.3 (7.2)			
Early-underweight	76.6 (6.9)			
On-time-obesity	77.4 (6.5)			
On-time-overweight	77.3 (6.5)			
On-time-healthyweight	77.3 (5.3)			
On-time-underweight	76.25 (6.4)			
Late-obesity	77.4 (6.5)			
Late-overweight	77.3 (6.5)			
Late-healthyweight	77.3 (5.3)			
Late-underweight	76.25 (6.4)			

IQR, interquartile range; *p*_1_ value, significance test result between maturity groups; *p*_2_ value, significance test result between obesity groups; *p*_3_ value, the results of the in-group comparison significance test.

**Figure 5:**
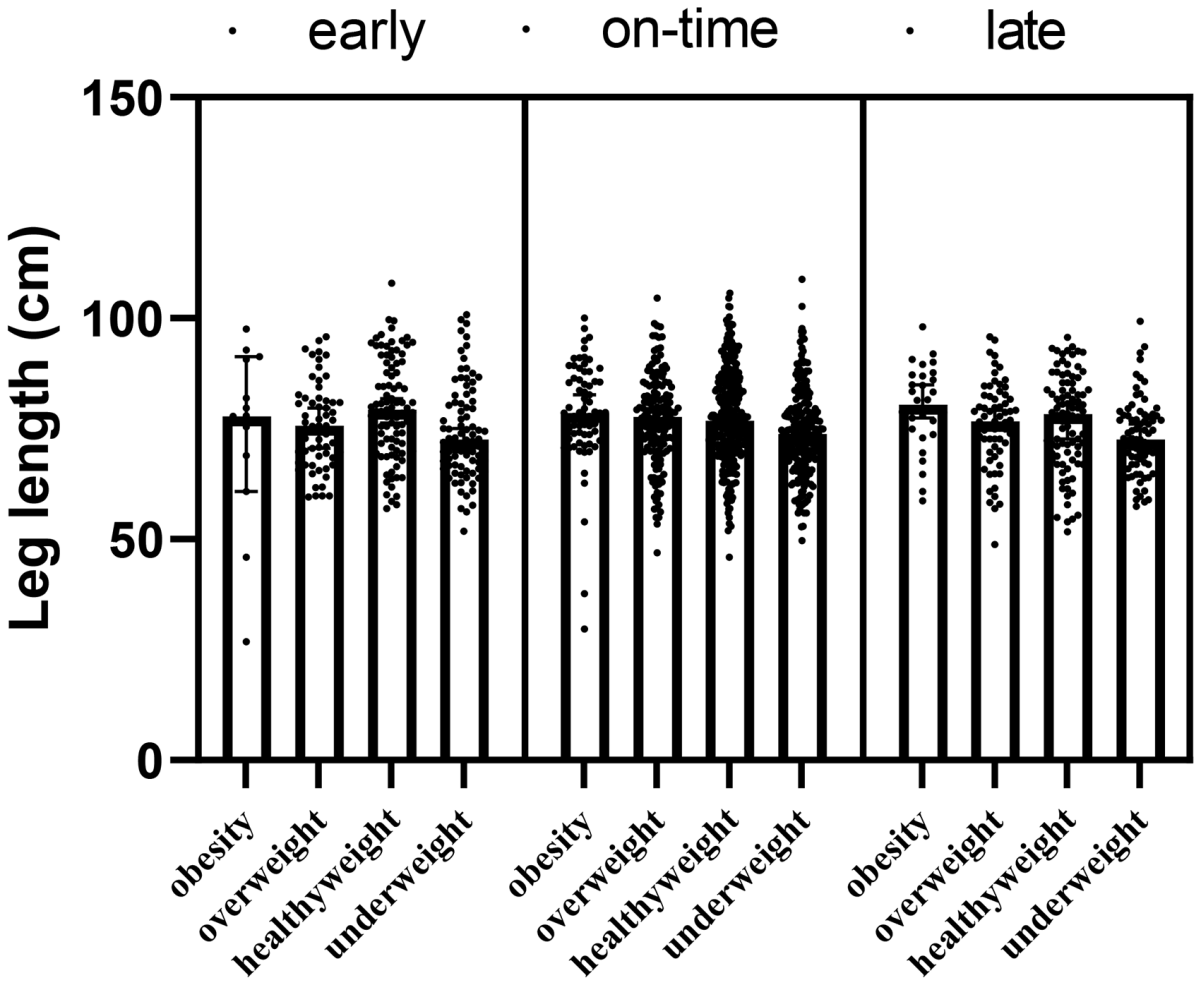
The leg length according to maturity and obesity groups of participants.

The main effect of maturity (*F* = 0.352; *p*_1_ = 0.543) and the interaction effect of maturity x obesity (*F* = −87.313; *p* = 0.165) were not detected in the leg length results, but the main effect of obesity was significant for leg length (*F* = 6.825; *p*_2_ < 0.001). The obesity, overweight and healthy weight group had a significantly higher leg length compared to the underweight group (*p* < 0.05) (Table 5; Figure 5).

**Table 5:** Changes in the leg length of the participants according to the maturity and obesity groups.

Groups	Median (IQR)	Maturity main effect	Obesity main effect	Interaction
		*F* value	*F* value	
		*p*_1_ value	*p*_2_ value	
Early-obesity	77.85 (21.8)	*F* = 0.352 *p*_1_ = 0.543	*F* = 6.825 *p*_2_ < 0.001	*F* = −87.313 *p* = 0.165
Early-overweight	75.7 (14.6)			
Early-healthyweight	79.3 (19.1)			
Early-underweight	72.55 (14.5)			
On-time-obesity	78.6 (14.1)			
On-time-overweight	77.7 (13.3)			
On-time-healthyweight	76.8 (14)			
On-time-underweight	73.8 (11.7)			
Late-obesity	78.6 (14.1)			
Late-overweight	77.7 (13.3)			
Late-healthyweight	76.8 (14)			
Late-underweight	73.8 (11.7)			
*Post-hoc* analysis for obesity factor		Obesity-underweight *p*_3_ < 0.001		
		Overweight-underweight *p*_3_ < 0.001		
		Healthyweight-underweight *p*_3_ < 0.001		

IQR, interquartile range; *p*_1_ value, significance test result between maturity groups; *p*_2_ value, significance test result between obesity groups; *p*_3_ value, the results of the in-group comparison significance test.

Age, weight, height, sitting height, leg length, chronological age, sex and obesity were included in the model as predictive variables in binary logistic regression analysis. Age, weight, height, chronological age, sex, and obesity had significant OR *p* values for maturity. It was determined that one unit increase in age increased early maturation 7.94 times. Chronological age was an enhancing predictor for early maturation, and the risk of premature maturation was 1,000, 76.92, 5.35 fold higher in the 10, 11, and 12 chronological age groups, respectively, compared to the 13 year age group. Furthermore, boys matured 3.077 fold later than girls. Therefore, being a girl was an enhancing predictor for early maturation. Weight was an important determinant for maturity groups, and a one-unit increase in weight increased late maturity 1.098 fold. Height showed a enhancing effect on early maturation, and it was determined that an increase in height by one unit increased early maturation 1.15 fold. Obesity had an increasing effect on early maturation. It was determined that being obese, overweight and healthyweight increased the risk of early maturation 9.80, 6.99 and 1.81 fold, respectively. As a result, The equation of the model predicting maturation is: Logit (*P*) = 1/(1+ exp. (− (−31.386 + boy * (1.124) + [chronological age = 10] * (−7.031) + [chronological age = 11] * (−4.338) + [chronological age = 12] * (−1.677) + age * (−2.075) + weight * 0.093 + height * (−0.141) + obesity * (−2.282) + overweight * (−1.944) + healthy weight * (−0.592)))).

With the developed logistic regression-based equation, it can be quickly determined whether a person matures early or late (Table 6).

**Table 6:** Logistic regression results.

Maturity	B	SE	Wald	Value of *p*	OR	95% CI for OR	
						Lower bound	Upper bound
[Sex = boy]	1.124	0.279	16.215	**<0.001**	3.077	1.780	5.318
[Chronological age = 10]	−7.031	0.877	64.227	**<0.001**	0.001	0.001	0.005
[Chronological age = 11]	−4.338	0.604	51.627	**<0.001**	0.013	0.004	0.043
[Chronological age = 12]	−1.677	0.382	19.304	**<0.001**	0.187	0.089	0.395
[Chronological age = 13]			71.343	**<0.001**			
Age (years)	−2.075	0.256	65.645	**<0.001**	0.126	0.076	0.207
Weight (kg)	0.093	0.029	10.093	**0.001**	1.098	1.036	1.163
Height (cm)	−0.141	0.063	5.028	**0.025**	0.869	0.768	0.982
Sitting height (cm)	0.063	0.061	1.069	0.301	1.065	0.945	1.201
Leg length (cm)	0.063	0.059	1.162	0.281	1.065	0.950	1.195
Obesity	−2.282	1.047	4.751	**0.029**	0.102	0.013	0.794
Overweight	−1.944	0.665	8.553	**0.003**	0.143	0.039	0.527
Healthy weight	−0.592	0.321	3.404	0.065	0.553	0.295	1.038
Underweight			9.181	**0.027**			
Constant	31.386	4.007	61.354	**<0.001**			

B, coefficient; SE, standard error; OR, odds ratio; CI, confidence interval; bold indicate statistically significant.

Table 7 shows the performance criteria results and confidence intervals regarding the estimation performance of the logistic regression model. Our model predicted maturity with 80.7% [95% CI: 77.2–84.1%] accuracy. In addition, the model had a high sensitivity value (81.7% [76.2–86.6%]), which indicates that the model can successfully distinguish adolescents with early maturation.

**Table 7:** Results of performance metrics for Lojistic regression model maturity prediction.

Metric	Value	95% CI lower limit	95% CI upper limit
Accuracy	0.807	0.772	0.841
F1-score	0.795	0.760	0.830
Sensitivity	0.817	0.762	0.864
Specificity	0.798	0.746	0.844
Positive predictive value	0.774	0.717	0.825
Negative predictive value	0.837	0.787	0.880

CI, confidence interval.

## 4. Discussion

The primary aim of this study was to examine the relationship between biological maturation and obesity. To the best of our knowledge, this is the first study predicting maturity by measuring some obesity parameters based on machine learning approach. Our predictive model showed that obesity increases the risk of early maturation. In addition, early maturation was higher in adolescent girls. Our model predicted maturity with 80.7% [95% CI: 77.2–84.1%] accuracy. Moreover, the high sensitivity value of the model (81.7% [76.2–86.6%]) indicates that the model can successfully distinguish early maturing adolescents. Consistent with the current study, it was found in a study that obesity is associated with maturation in both boys and girls (1,525 boys and 1,501 girls aged 8–14), however, the association was differed.

Although a positive correlation was found in girls which is consistent with our study, a negative one was reported in boys unlike the present study (39). This controversial results would be attributed to the study design, evaluation technique different populations studied and study duration. In justifying the obtained results for boys, it should be noted that there are conflictary results regarding the relationship between obesity and timing of pubertal onset in boys (40, 41) and this can be a reason for low correlation of obesity and maturity in boys rather than girls. In other words, obesity would contribute to early onset of puberty in girls more than boys. One possible reason for this effect refers to higher threshold of BMI for puberty development in boys than girls (41). The second aim was to examine the effects of anthropometric measurements, gender, and obesity on biological maturation in adolescents. Our results showed that the maturity*obesity interaction effect was significant for age, body weight, and height. Age, weight, height, chronological age, gender, and obesity were important risk factors and predictors for maturity. Interestingly, in a logitudinal study lasting more than 10 years, it was reported that pubertal growth patterns, including earlier puberty onset timing, smaller puberty intensity, and shorter puberty spurt duration, had a positive association with higher obesity risks in late adolescence (42). Notably, contraversial studies in boys are more observed compared to the girls. For instance, earlier puberty was found in overweight boys compared to normal weight and later puberty in obese compared to overweight (4,131 boys from 2005–2010) (43). In a study, it was shown that with each unit increase in childhood BMI, the age of peak height velocity (PHV) was earlier by 2 months just in normal weight boys, while the same was not found in overweight ones (6). In agreement with our study, there are several studies demonstrating the positive correlation between body composition and earlier onset of puberty in obese girls (44, 45). Based on research evidences, there are some reasons for the early onset of puberty in girls including endocrine-disrupting chemicals (46), psychosocial factors (47), and chronic stress (48). One possible mechanism involved in earlier puberty would be related to sex hormones changes. For instance, Increased levels of estradiol secretion would cause some changes in body fat distribution in pubescent girls. Accordingly, high estradiol levels are associated with precocious puberty (49). In reality, the complicated etiology of obesity is influenced by a variety of variables, including biological, behavioral, environmental, physical activity and genetic ones (8, 50). A wide range of exercise-related health advantages may be promoted by studying the lipidomic profile, which enables complicated biological processes to be adjusted more efficiently in the context of exercise (51). Physical exercise has numerous repercussions on metabolism and function of different organs and tissues by enhancing whole-body metabolic homeostasis in response to different exercise-related adaptations. In a review, Latino et al. showed as exercise determine significant changes in lipidomic profiles, but they manifested in very different ways depending on the type of tissue examined (52) As healthy lifestyle during pubertal years is highly related to accelerated biological maturation in childhood and adolescence (53, 54), its recommended to consider it in future studies.

There are several limitations in the study. One of the main limitations of this study is that it is a cross-sectional study. The second one refers to the lack of measurement of testicular enlargement as a classic marker of pubertal onset, since it requires invasive palpation. It should be also noted that there are limited reliable self-reported markers for pubertal timing in boys and most data rely solely on visual grading of genital development. Another limitation of this study is the impossibility of laboratory measurements such as sex hormone levels and thelarche evaluation, which is recommended in future studies.

## 5. Conclusion

Today, the use of machine learning technology is one of the advantages that allows the prediction of scientific facts in the best possible way. In conclusion, sex and obesity are independent predictors of maturity, and the risk of early maturation is increased, especially in the case of obesity in girls. This study’s main conclusion was that early biological maturation was strongly associated with obesity, particularly in females. In other words, early biological maturation in girls may result in their going through the menstrual cycle early and, as a result, developing a number of health issues as adults. Preventive programs can aid in their timely entry into the biological maturation process by further examining the fundamental mechanism of obesity. The participation of several public and private partners is necessary to stop the children and adolescent obesity epidemic and reduce the health hazards related to obesity. Governments, international partners, non-governmental organizations, and the corporate sector all have a crucial role to play in encouraging physical activity and better nutrition for children and adolescents.

## Data availability statement

The raw data supporting the conclusions of this article will be made available by the authors, without undue reservation.

## Ethics statement

The studies involving human participants were reviewed and approved by The study was approved by Kirikkale University Non-Interventional Research Ethics Committee (date: 2022-06-08, no: 2022/10) and was conducted according to the principles stated in the Declaration of Helsinki. Written informed consent to participate in this study was provided by the participants’ legal guardian/next of kin.

## Author contributions

MG and FY: conceptualization, validation, and supervision. MG, HY, and FY: methodology and investigation. FY: software, formal analysis, resources, and data curation. MG, HY, AD, KI, and MT: writing – original draft preparation. MG, KI, MT, TG, MB, and ES: writing – review and editing. AD: visualization. MG: project administration. MB, ES, and TG: funding acquisition. All authors contributed to the article and approved the submitted version.

## Funding

Published with the financial support of the European Union, as part of the project entitled Development of capacities and environment for boosting the international, intersectoral, and interdisciplinary cooperation At UWB, project reg. no.CZ.02.2.69/0.0/0.0/18_054/0014627.

## Conflict of interest

The authors declare that the research was conducted in the absence of any commercial or financial relationships that could be construed as a potential conflict of interest.

## Publisher’s note

All claims expressed in this article are solely those of the authors and do not necessarily represent those of their affiliated organizations, or those of the publisher, the editors and the reviewers. Any product that may be evaluated in this article, or claim that may be made by its manufacturer, is not guaranteed or endorsed by the publisher.
